# Reply to Comment on “Kadomoto, S. et al. Tumor-Associated Macrophages Induce Migration of Renal Cell Carcinoma Cells via Activation of the CCL20-CCR6 Axis” *Cancers* 2020, *12*, 89

**DOI:** 10.3390/cancers12020354

**Published:** 2020-02-04

**Authors:** Suguru Kadomoto, Kouji Izumi, Kaoru Hiratsuka, Taito Nakano, Renato Naito, Tomoyuki Makino, Hiroaki Iwamoto, Hiroshi Yaegashi, Kazuyoshi Shigehara, Yoshifumi Kadono, Hiroki Nakata, Yohei Saito, Kyoko Nakagawa-Goto, Atsushi Mizokami

**Affiliations:** 1 Department of Integrative Cancer Therapy and Urology, Kanazawa University Graduate School of Medical Science, Kanazawa 920-8641, Japan; 32f3k8@bma.biglobe.ne.jp (S.K.); hirakaoru@med.kanazawa-u.ac.jp (K.H.); urotaito@yahoo.co.jp (T.N.); thealfuu@yahoo.co.jp (R.N.); mackeeen511@gmail.com (T.M.); hiroaki017@yahoo.co.jp (H.I.); hyae2002jp@yahoo.co.jp (H.Y.); kshigehara0415@yahoo.co.jp (K.S.); yskadono@yahoo.co.jp (Y.K.);; 2 Department of Histology and Cell Biology, Kanazawa University Graduate School of Medical Science, Kanazawa 920-8641, Japan; hnakata@staff.kanazawa-u.ac.jp; 3 School of Pharmaceutical Sciences, College of Medical, Pharmaceutical and Health Science, Kanazawa University, Kanazawa 920-1192, Japan

We appreciate Zins and Abraham [1] commenting on our paper studying the role of the CCL20-CCR6 axis on renal cell carcinoma (RCC) cells [2]. As they pointed out, our study has certain limitations. Although M1- and M2-types cannot be separated clearly and a consecutive change of character might exist between them, it has been reported that plural specific markers express on M1- and M2-types. Unfortunately, a definite difference between M1 and M2 macrophages was not confirmed in our study. For more differentiation, multiple stimulations, such as suggested in the comments of Zins and Abraham, might be needed. Hence, we needed to expediently use “M1-like” and “M2-like” to mention specific status of these macrophage-like cells. Meanwhile, CCL20 expression levels of M2-like-THP-1 cells co-cultured with RCC cells were dramatically increased compared with parental THP-1 cells, indicating that certain stimulations within the tumor microenvironment rather than theoretical stimulations make macrophages differentiated; however, further studies are needed to clarify this mechanism using a more appropriate co-culture system mimicking the tumor microenvironment. Immunohistochemistry of CCL20 and M2 markers will help to better understand the role of tissue infiltrating macrophages, even tissue CD68 staining intensity itself was reported to correlate with prognosis of RCC patients [3]. As, CCL20, a secreted protein, is distributed diffusely in tissue, the identification of cells secreting CCL20 might be challenging. Instead of CCL20 staining, we performed CCR6 staining to depict the functional effect of CCL20. We agree that distinct staining of M1 and M2 markers in RCC tissue with increased sample number and the use of patient-derived M2 macrophages from RCC patients will clarify the role of the CCL20-CCR6 axis on RCC progression.
